# Comparison of different annotation tools for characterization of the complete chloroplast genome of *Corylus avellana* cv Tombul

**DOI:** 10.1186/s12864-019-6253-5

**Published:** 2019-11-20

**Authors:** Kadriye Kahraman, Stuart James Lucas

**Affiliations:** 1 Faculty of Engineering and Natural Sciences, Sabanci University, 34956 Istanbul, Turkey; 2 Sabanci University Nanotechnology Research and Application Centre (SUNUM), Sabanci University, 34956 Istanbul, Turkey

**Keywords:** *Corylus avellana*, Tombul cultivar, Hazelnut, Chloroplast genome, Phylogeny

## Abstract

**Background:**

Several bioinformatics tools have been designed for assembly and annotation of chloroplast (cp) genomes, making it difficult to decide which is most useful and applicable to a specific case. The increasing number of plant genomes provide an opportunity to accurately obtain cp genomes from whole genome shotgun (WGS) sequences. Due to the limited genetic information available for European hazelnut (*Corylus avellana* L.) and as part of a genome sequencing project, we analyzed the complete chloroplast genome of the cultivar ‘Tombul’ with multiple annotation tools.

**Results:**

Three different annotation strategies were tested, and the complete cp genome of *C. avellana* cv Tombul was constructed, which was 161,667 bp in length, and had a typical quadripartite structure. A large single copy (LSC) region of 90,198 bp and a small single copy (SSC) region of 18,733 bp were separated by a pair of inverted repeat (IR) regions of 26,368 bp. In total, 125 predicted functional genes were annotated, including 76 protein-coding, 25 tRNA, and 4 rRNA unique genes. Comparative genomics indicated that the cp genome sequences were relatively highly conserved in species belonging to the same order. However, there were still some variations, especially in intergenic regions, that could be used as molecular markers for analyses of phylogeny and plant identification. Simple sequence repeat (SSR) analysis showed that there were 83 SSRs in the cp genome of cv Tombul. Phylogenetic analysis suggested that *C. avellana* cv Tombul had a close affinity to the sister group of *C. fargesii* and *C. chinensis*, and then a closer evolutionary relationship with Betulaceae family than other species of Fagales.

**Conclusion:**

In this study, the complete cp genome of *Corylus avellana* cv Tombul, the most widely cultivated variety in Turkey, was obtained and annotated, and additionally phylogenetic relationships were predicted among Fagales species. Our results suggest a very accurate assembly of chloroplast genome from next generation whole genome shotgun (WGS) sequences. Enhancement of taxon sampling in *Corylus* species provide genomic insights into phylogenetic analyses. The nucleotide sequences of cv Tombul cp genomes can provide comprehensive genetic insight into the evolution of genus *Corylus*.

## Background

European hazel (*Corylus avellana* L.) is a crop tree of worldwide agronomic importance, which has been cultivated for human consumption for thousands of years with a large geographic distribution [1]. Hazelnuts are high in unsaturated fats and contain many essential and minerals, and thereby *C. avellana* occupies an important place in human nutrition [2]. Broad usage of *C. avellana*, such as adding flavor and texture to dairy, bakery, confectionary and chocolate products, indicate its value to the food industry. Even though it has a significant place in agriculture, a limited number of studies exists about *C. avellana* at the molecular level. Currently the only available genome sequences for *C. avellana* is a draft genome for the American cultivar ‘Jefferson’ [3]. In this study, we report the chloroplast genome sequences of Tombul cultivar, the most widely grown Turkish variety, from next generation whole genome shotgun sequences.

The chloroplast (cp) is the main site of photosynthesis and contains enzymatic mechanisms for carbohydrate biosynthesis. The cp genomes of plants are highly conserved in terms of gene size, content and organization, and have a simple circular, quadripartite structure, including two copies of an inverted repeat (IR) that separate the large and small single copy regions (LSC and SSC). Because of its conserved nature, the cp genome contributes to plant systematics and evolutionary studies [4–6]. In addition, due to their small genome size, it is much easier to compare cp genomes than the whole genomic data for genomic comparative analysis. Early on chloroplast DNA (cpDNA) fragments are often used as ‘DNA barcodes’ in inter-species phylogenetic analysis due to their universal presence and abundance in plant cells. However, Yang et al. [7] indicated that the cpDNA fragments most commonly used in phylogenetic analysis such as *matK*, *rbcL* and *trnH*-*psbA*, have little sequence divergence in genus *Corylus*, thus it is hard to precisely resolve phylogenetic relationships within the genus using these fragments. Especially in the phylogeny of land plants, studies demonstrated that complete chloroplast genomes provide more reliable information than cpDNA barcode sequences, and eliminate problems associated with barcoding, such as primer design and amplification [8–12]. The complete cp genomes are useful and cost-effective for resolving phylogenetic relationship at both high and low taxonomic levels because they contain both conserved and variable protein-coding genes; also, compared to the nuclear genome cp genomes exhibit a slower evolutionary rate and mostly uniparental inheritance [13–19]. Limited sequence variation has led to the use of cp genomes mostly in studies at the interspecific and interfamilial levels [13, 14, 20, 21]. In addition, cp genomes provide deeper information for phylogeny reconstruction of *Corylus* species in comparison with previous studies that relied on molecular markers, including RAPD [22], SSR [23, 24], SRAP [25], ISSR [26, 27], AFLP [28], and DNA fragments such as ITS regions and cpDNA fragments [28–30]. The whole cp genome is also useful for identification of plant varieties by allowing selection of highly variable non-genic markers for DNA barcoding [31, 32].

Barker et al. [33] indicate that next generation whole genome shotgun (WGS) sequences from plants typically contain 5% or more reads derived from the chloroplast. Thus, the sequenced genome data of plant species can be used to obtain cp genomes without prior isolation of cpDNA. Due to the development of next generation sequencing technology, an increasing number of WGS datasets are available for cp genome assembly. Wang et al. [34] revealed the complete cp genomes of *Fagopyrum dibotrys* from high-throughput sequencing datasets, and obtained reliable chloroplast genomes. Osuna-Mascaró et al. [35] also retrieved the cp genome of *Erysimum* (Brassicaceae) species from a genomic library, and achieved similar cp genomes in terms of overall size, structure and composition. Besides de novo assembly of complete chloroplast genome, alignment-based methods can also be used to obtain cp assemblies from WGS reads by mapping them onto a reference cp genome [36]. However, this latter method relies on the availability of a high quality cp genome from a related species.

Herein, we present the complete cp genome of *Corylus avellana* cv Tombul. The aim of the study was to compare different available annotation tools, develop an optimized pipeline for cp assembly and annotation form WGS sequences, and examine the cp genome structure, gene content and gene order of Turkish hazelnut. Although there is a chloroplast genome for *C. avellana* in NCBI (KX822768), there is no detailed information about the construction of this genome or which variety of hazelnut it originates from. Therefore, we chose to generate a new annotation for one of the most commercially important Turkish hazelnut cultivars, ‘Tombul’. Moreover, simple sequence repeats (SSRs) are investigated in cv Tombul cp genome, and phylogenetic relationships are predicted among the Fagales, including genera Betulaceae, Fagaceae and Juglandaceae.

## Results

### Size, gene content, order and organization of the hazelnut

Initial assembly using the NOVOplasty assembler with raw *C.avellana* cv ‘Tombul’ WGS sequences produced a single 200,017 bp contig [37]. The length of this contig was significantly longer than the *C. avellana* cp genome previously published in GenBank (Accession no: KX822768). Therefore, the raw contig was aligned to the KX822768 cp genome, and it was observed that the last part, starting from 161,667 bp, consisted of repeats of sequences from the rest of the Tombul chloroplast genome. To demonstrate whether the extra part, located after 161,667 bp, was genuine or not, Nanopore sequencing reads belonging to cv Tombul were also aligned to the contig. Although a subset of reads matched these additional parts in two segments, the mapped read depth of the these segments was approximately half of that of the rest of the cp genome. Moreover, BLAST alignment found that the additional part was 100% identical to two regions in the first 161 kb of the cv Tombul cp genome [38]. These observations suggested that the extra 39 kb in our initial contig was an artefact of the NOVOplasty assembly algorithm, where the duplicated segments were incorporated twice, perhaps due to sequence variation at their boundaries.

In addition, we examined whether a single circular cp genome could be retrieved using a standard whole genome assembly algorithm, rather than one specific to the chloroplast. For this test, trimmed WGS sequences were assembled using ABySS assembler [39], and then the cv Tombul cp genome constructed by NOVOplasty and the KX822768 cp genome were mapped to these contigs of cv Tombul genome using BLAST. Multiple contigs from the whole genome assembly matched the chloroplast sequences, but they were overlapping and fragmented (data not shown). Therefore it was concluded that using an assembler specialized for organellar genomes is advantageous for cp genome construction; further analysis was carried out using the first 161,667 bp of the genome assembly obtained from NOVOplasty, which also showed high similarity to the KX822768 cp genome.

**Fig. 1:**
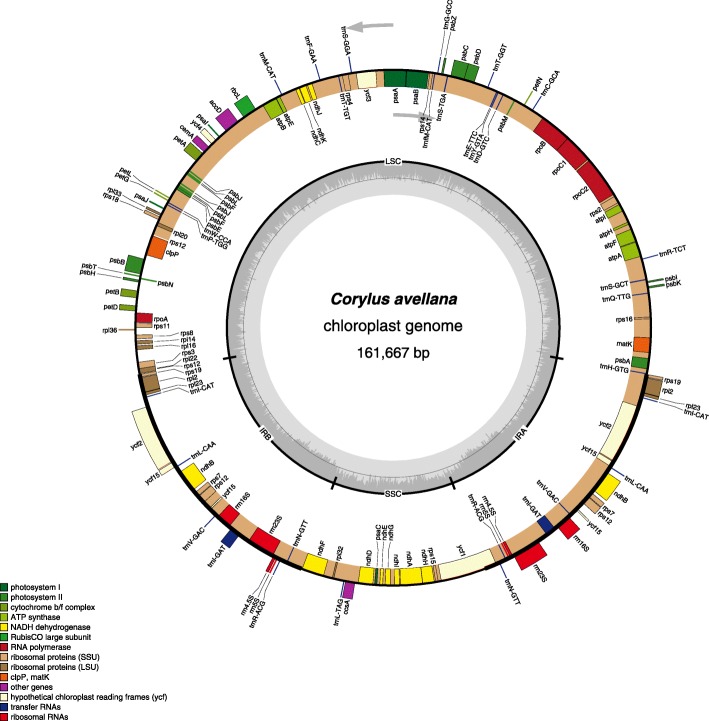
The chloroplast genome map of *Corylus avellana* cv Tombul species. Genes lying outside the circle are transcribed in the counter clockwise direction, while those inside are transcribed in clockwise direction. The colored bars indicted different functional groups. The darker gray area in the inner circle denotes GC content while the lighter gray corresponds to the AT content of the genome. LSC, large single copy; SSC, small single copy; IR, inverted repeat

The Tombul complete cp genome had a length of 161,667 bp and includes a pair of inverted repeats 26,368 bp long, separated by a small and a large single copy region of 18,733 bp and 90,198 bp, respectively (Fig. 1). The overall GC content of cv Tombul cp genome was 36.40%, and GC contents of the LSC and the SSC regions were 34.17 and 30.25%, respectively. The GC content of the IR region was much higher than that of the LSC and SSC regions with 42.37%, due to its relatively abundant GC-rich tRNA and rRNA genes.

**Fig. 2:**
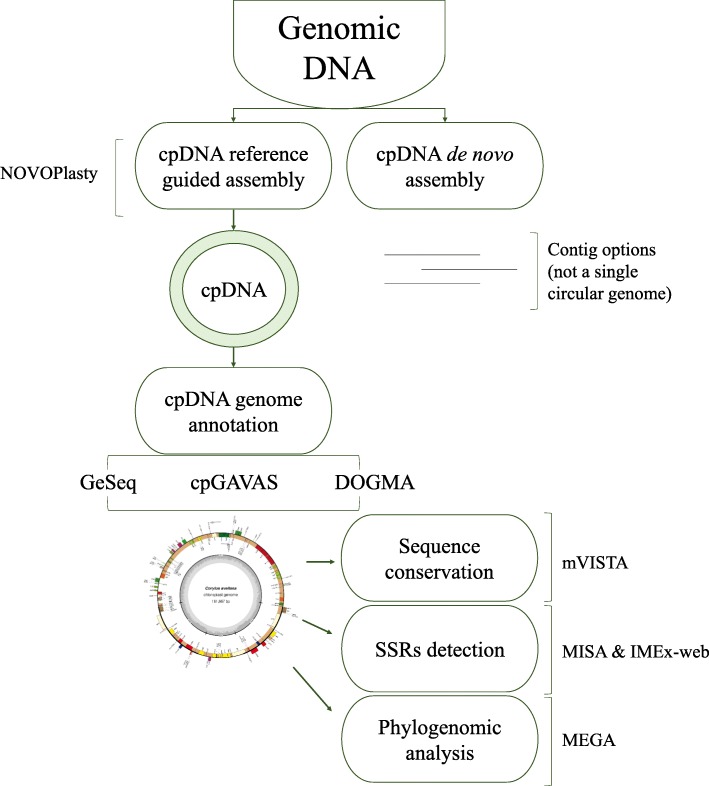
Sequence alignment of 8 chloroplast genomes using mVISTA tool with *Corylus avellana* cv Tombul as a reference. Grey arrows above the alignment indicate the transcriptional directions of genes. Genome regions, exon and conserved non-coding sequences (CNS), are color coded as blue and red, respectively. Multiple alignment was carried by LAGAN option, and a cut-off of 50% identity was used for the plots. The Y-axis indicated the percent identity between 50 and 100%

For annotation of functional genes, three different prediction tools, namely GeSeq, cpGAVAS, and DOGMA, were compared (Fig. 2). These agreed with each other for the majority of the content and order of genes [40–43]. Generally, genes were included in the final map when at least 2 of the tools gave matching predictions. A total of 125 predicted functional genes were encoded within the *Corylus avellana* cv Tombul cp genome. Among them, 88 genes were unique, while 17 genes were duplicated in the IR region (IRA and IRB). Furthermore, the 105 distinct genes comprised 76 protein-coding, 25 tRNA and 4 rRNA genes. Seven protein coding genes (*ndhB, rpl2, rpl23, rps7, rps12, rps19,* and *ycf2*), six of the tRNA genes (*trnI-CAT, trnI-GAT, trnL-CAA, trnN-GTT, trnR-ACG,* and *trnV-GAC*) and all rRNA genes (*rrn16, rrn23, rrn5* and *rrn4.5*) were duplicated within the IR. Although the 3 annotation tools gave similar gene predictions a few differences were detected, especially in tRNA genes. The genes for *trnA-TGC* (duplicated in IR), *trnK-TTT, trnL-TAA* and *trnV-TAC* were only annotated by DOGMA, therefore they were not included in the final map. Fifty seven protein-coding genes and 18 tRNA genes were contained in the LSC region, while 12 protein-coding genes and one tRNA gene were identified in the SSC region. Three open reading frames (*orf42*, *orf56*, and *orf188*) and an addition hypothetical chloroplast reading frame (*ycf68*) were also identified with the DOGMA tool. Moreover, one gene, *ycf1* located in the IRA/SSC junction, extended the IRA region by several bases. A *ycf*-like gene was also reported in the IRB region, one of the two IRs, with two annotation tools, DOGMA and GeSeq, but it was a truncated fragment of *ycf1* gene, and thus not included in the genome map. Of the 76 unique protein-coding genes, five genes (*atpF*, *ndhA*, *ndhB*, *rpl2*, and *rpoC1*) contained one intron, while two protein-coding genes (*clpP* and *ycf3*) contained two introns each. The gene *rps12* was annotated as trans-spliced gene of which the 5′-end exon was located in the LSC region while its intron and 3′- end exon were situated in the IR region (Additional file 1: Tables S1, S2).

RNA editing, a post-transcriptional modification process, exists in chloroplasts to encode appropriate amino acids and maintain conserved protein functions by correcting codons, especially by alteration of nucleotides from cytosine to uracil (C-to-U) and less frequently from uracil to cytosine (U-to-C) [44–46]. Wang et al. [47] indicated that several changes were observed in protein-coding transcripts from chloroplasts, including C to U, along with G to A and C to G, A to G and G to A. Several nucleotide alterations are required to provide functional start codons in a handful of the genes annotated in the present study (Additional file 1: Table S3). RNA editing at these sites has not previously been confirmed in the Betulaceae, thereby further RNA sequence analysis should be carried out to determine whether these modifications occur.

Comparing the results of the annotation tools, ten genes (*atpF*, *clpP*, *ndhA*, *ndhB*, *ndhK*, *petA*, *rpl2*, *rpoC1*, *ycf3*, *ycf15*) were erroneously reported twice as 2 gene fragments by DOGMA and GeSeq, whereas they were correctly reported as a single gene containing an intron by cpGAVAS (Additional file 1: Table S9). When the annotated genes were compared with those previously reported in other species’ chloroplast sequences, the GeSeq tool gave the most accurate results for gene locations, including starting and end points of the CDS. DOGMA did not define the start and end point of exons, therefore start and stop codons had to be manually checked, and added from the cp genome. All of the genome and annotation information is shown in Fig. 1.

Prediction of cv Tombul cp gene functions was based on homology, and as expected they were mostly involved in photosynthesis and other metabolic processes. The genes were classified into three broad categories based on their functions: photosynthesis, self-replication and other genes. While 42 protein-coding genes participated in photosynthesis, 25 protein-coding genes were involved in the chloroplast self-replication processes, and 5 genes represented other functions, all of which were summarized in Table 1.

**Table 1:** Gene contents and functional classification of cv Tombul chloroplast genome

Category	Group of genes	Code of genes	List of genes
Genes for photosynthesis	Subunits of ATP synthase	atp	atpA, atpB, atpE, atpF, atpH, atpI
	Subunits of NADH-dehydrogenase	ndh	ndhA, ndhB, ndhC, ndhD, ndhE, ndhF, ndhG, ndhH, ndhI, ndhJ, ndhK
	Subunits of cytochrome b/f complex	pet	petD, petG, petL, petN
	Subunits of photosystem I	psa	psaA, psaB, psaC, psaI, psaJ
	Subunits of photosystem II	psb	psbA, psbB, psbC, psbD, psbE, psbF, psbH, psbI, psbJ, psbK, psbL, psbM, psbN, psbT, psbZ
	Subunit of rubisco	rbc	rbcL
Self-replication	Large subunit of ribosome	rpl	rpl2,rpl14,rpl16, rpl20, rpl22, rpl23, rpl32, rpl33, rpl36
	DNA dependent RNA polymerase	rpo	rpoA, rpoB, rpoC1, rpoC2
	Small subunit of ribosome	rps	rps2, rps3, rps4, rps7, rps8, rps11, rps12, rps14, rps15, rps16, rps18, rps19
	rRNA Genes	rrn	rrn4.5S, rrn5S, rrn16S, rrn23S
	tRNA Genes	trn	trnC-GCA, trnD-GTC, trnE-TTC, trnF-GAA, trnfM-CAT, trnG-GCC, trnH-GTG, trnM-CAT, trnP-TGG, trnQ-TTG, trnR-TCT, trnS-GCT, trnS-GGA, trnS-TGA, trnT-GGT, trnT-TGT, trnW-CCA, trnY-GTA, trnL-TAG, trnI-CAT, trnI-GAT, trnL-CAA, trnN-GTT, trnR-ACG, trnV-GAC
Other genes	Subunit of Acetyl-CoA-carboxylase	acc	accD
	c-type cytochrome synthesis gene	ccs	ccsA
	Envelop membrane protein	cem	cemA
	Protease	clp	clpP
	Maturase	mat	matK
Genes of unkown function	Conserved open reading frames	ycf	ycf1, ycf2, ycf3, ycf4

Based on a sequence similarity search of the whole genome, the *C. avellana* cv Tombul chloroplast was most similar to chloroplast genomes belonging to the *Corylus* family with a range from 99.46 (*Corylus wangii*, Accesion: MH628454.1) to 99.88% (*Corylus heterophylla* var. sutchuenensis, Accesion: MF996573.1) identity via Basic Local Alignment Search Tool (BLAST) search in NCBI website (http://blast.ncbi.nlm.nih.gov/) against Viridiplantae (taxid: 33090) [38]. In addition, *Carpinus* and *Ostrya* families also showed high similarity with cv Tombul cp genome with nearly 98.91 and 99.21% identity, respectively. (Additional file 1: Table S4).

### Comparison of chloroplast genome sequences with other species

**Fig. 3:**
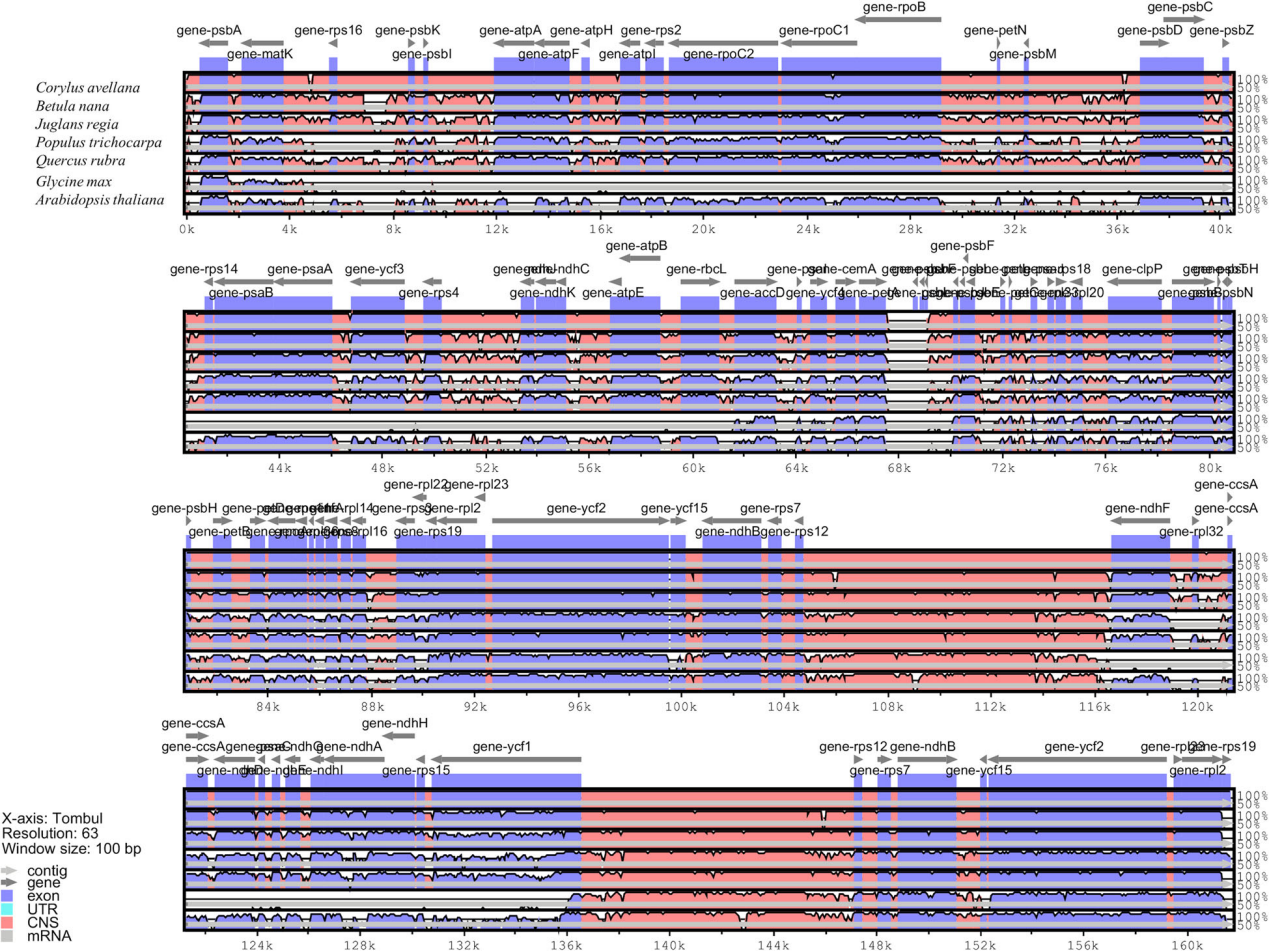
Phylogenetic position of *Corylus avellana* cv Tombul inferred by maximum likelihood (ML) analysis of 22 complete cp genomes. Numbers above each node indicate the bootstrap values based on 500 replicates

The similarities and differences of the cp genome between *C. avellana* cv Tombul and other species, including representatives of the Malpighiales, Fabales and Brassicales, were determined by a global alignment program, mVISTA [48]. The chloroplast genome sequences were aligned to each other and plotted using *C. avellana* cv Tombul as a reference (Fig. 3). Tombul had a similar cp genome size to the other species, which range from 152,217 bp to 161,303 bp (Tombul cp genome size is 161,667 bp). In addition, the alignment revealed a very high level of identity in the global patterns of sequence similarities with KX822768, an accession of an unspecified *C. avellana* variety found in China, and *Betula nana* with 99.8 and 96.6% identity, respectively. As expected, coding regions were more highly conserved than non-coding regions. The highest polymorphism was observed in intergenic regions (such as *rps16*-*psbK*, *psbI*-*atpA*, *psbM*-*psbD*), but the *ycf1* gene had higher variability regions, especially between distant species. At the species level, nucleotide substitution could more rapidly occur in intergenic regions, and these regions with high levels of divergence could have high potential for developing molecular markers for population genetic analysis between varieties. Furthermore, a region was detected in the cv Tombul cp genome from ~ 68 to 69 kb that was conserved with KX822768 but none of the other species presented in the global alignment. This region contained duplicates of the *psbF*, *psbJ* and *psbL* genes from the adjacent region, and an unprocessed *petA* gene. This could be a tandem duplication specific to the hazelnut lineage; further *Corylus* chloroplast genomes should be explored to determine whether it is found in other species from this genus.

### SSR analysis

Simple sequence repeats (SSR) are useful in characterization of genetic diversity. According to the MISA web tool, a total of 83 SSRs were identified in the cv Tombul cp genome [49]. Among these SSRs, there were 44, 19, 4, 13, 2, 1 for mono-, di-, tri-, tetra- and penta- nucleotide repeats, respectively (Additional file 1: Table S5). The largest proportion of simple repeats was classified as mononucleotides (48.2%). While most of the mononucleotides were composed of A/T (90.9%), most of the dinucleotides were AT/TA (84.2%) (Additional file 1: Figure S1). Similar results were obtained from IMEx-web server [50]. Only a few differences were shown in the direction of SSRs (Additional file 1: Table S6). These SSR regions may be useful in developing markers useful to elucidate genome evolution and chloroplast rearrangements among species.

### Phylogeny inference

**Fig. 4:**
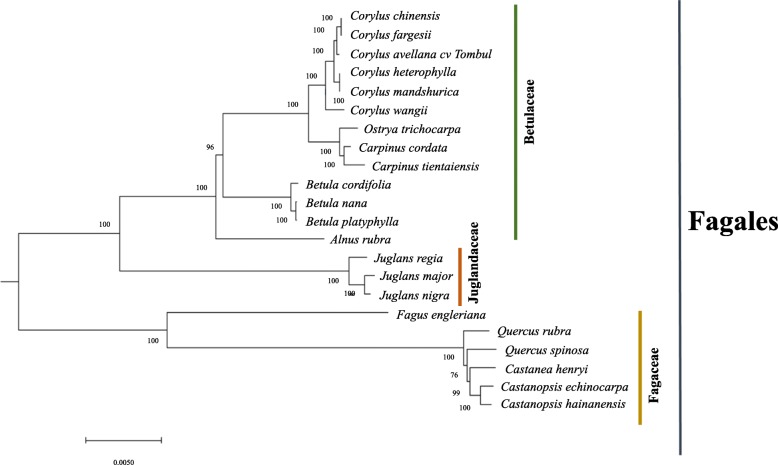
Flow chart describing the optimized bioinformatics pipeline for cp genome assembly

The complete cp genome sequences of 22 species from Fagales order were obtained from the NCBI and used for phylogenetic analysis, including representatives of genera of Betulaceae, Fagaceae, and Juglandaceae. As chloroplast protein sequences showed high similarity among related species, the phylogenetic analysis was carried out using the whole cp genome sequences. Tree construction was carried by the maximum likelihood method with 500 replicates. All nodes of these phylogenetic trees were strongly supported by bootstrap values (BS). The 22 taxa were classified into four major clades. A monophyletic group was observed incorporating the *Corylus*, *Betula* and *Juglans* species. Fagus and the sister group of *Quercus* and *Castanopsis* were located at the basal position. Moreover, within the Betulaceae, *Carpinus* and *Ostrya* clustered into a clade which was the sister to the clade *Corylus* and showed greater divergence from the clade formed by *Betula* species. As stated in the literature, *Corylus* was closest to *Carpinus* and *Ostrya* species, and then relatively close to *Betula*, which is consistent with their taxonomic classification but provides greater insight into the relatedness of these genera (Fig. 4) [51, 52].

In the clade *Corylus*, 6 species were divided into four subclades. *C. wangii* was located at the basal position, while *C. mandshurica* and *C. heterophylla* clustered into a sister group, while *C. fargesii* and *C. chinensis* clustered together. The phylogenetic tree indicated that cv Tombul, although it formed a distinct subclade, exhibited a closer relationship with *C. fargesii* and *C. chinensis* than the other varieties (Fig. 4) [53, 54].

## Discussion

### Comparison of methods for assembling cp sequences from WGS data

The assembly of cp genomes from whole genome shotgun (WGS) sequences is a useful strategy for characterizing cp genes, structure, function and phylogenetic relationships. Multiple tools have been developed to construct and annotate cp genomes. This study reported a complete cp genome sequence of *Corylus avellana* cv Tombul, annotated by different available annotation tools. Initially, the de novo assembler NOVOPlasty was used to reconstitute the Tombul cp genome (Fig. 2) [37]. A single 200,017 bp contig was obtained from raw WGS sequences by NOVOplasty. The comparison of the contig with the KX822768 cp genome, published in GenBank, indicated that the last part of the sequence, (161,667–200,017 bp), was nearly identical to other segments of the Tombul chloroplast genome. Nanopore sequencing reads belonging to cv Tombul, were aligned to the contig, and a subset of reads matched these additional parts. Therefore, we considered the possibility that the cp genome of cv Tombul could be physically larger than the reported *C. avellana* cp genome. However, BLAST results indicated that this part consisted of two segments, each of which was 100% identical to a region in the first 161 kb of the cv Tombul cp genome (Additional file 1: Figure S2) [47]. Furthermore, the mapped read depth of the duplicated segments was approximately half of that of the rest of the cp genome. Hence, we concluded that the additional 39 kb was an artefact of the NOVOplasty assembly algorithm. Further analysis was carried out using the first 161,667 bp of the genome assembly.

### Comparison of methods for annotation of cp genome for cv Tombul

The cv Tombul cp genome presented similar characteristics to other angiosperm cp genomes. In addition, it exhibited some differences between closely related species. There is a previously reported sequence for *C. avellana* deposited in Genbank (KX822768), cultivated in China, but no varietal information was provided for this accession. While the general characteristics of cv Tombul cp genome are highly consistent with KX822768, a few differences were detected at the gene level. Two genes, *atpF* and *clpP*, were reported as unprocessed in the older sequence, however full-length protein sequences were predicted for these genes in the cv Tombul cp genome. Furthermore, the genes *accD*, *psbM*, and *trnI*-*GAT*, were not annotated in KX822768, but they were present in the cv Tombul cp genome. Lastly, *psbF*, *psbJ* and *psbL* genes were found twice in the cv Tombul cp genome (Additional file 1: Table S7). The length of the cv Tombul cp genome was found to be similar to other *Corylus* and *Quercus* species, but a difference was indicated with *Populus* and *Juglans* species [53–57]. Although the length differed among species, the GC contents were very similar in angiosperm cp genomes (Additional file 1: Table S8) [58].

Two hypothetical chloroplast protein coding sequences, *ycf15* and *ycf68*, were also identified in our annotation process. Whereas they were represented as functional protein-coding genes in some studies [59], they were classified as pseudogenes in our map because of containing several internal stop codons in their coding sequences, consistent with observations in several other species [7, 60]. Moreover, the gene *ycf15* was annotated between *rps7* and *trnV*-*GAC* in some studies, while some others reported that it was located between *ycf2* and *trnL*-*CAA* [61, 62]. In the present study, the gene *ycf15* was detected in both positions, thereby further studies are required to clarify whether either of these is expressed.

The annotation of the cv Tombul cp genome was carried out using three different tools, cpGAVAS, DOGMA and GeSeq [40–43]. In terms of gene content, similar results were obtained from all tools. A number of genes were annotated as fragmented by DOGMA and GeSeq, whereas they were found as a single gene in cpGAVAS containing introns in specific locations. Additionally, more tRNA genes were detected using the DOGMA tool than the other two (Additional file 1: Table S9). The GeSeq annotation tool provides more precise results for gene locations, which may be because it chooses a broad range of BLAT reference sequences, including closely related taxa. Because it is not suitable for defining the start and end of exons, the DOGMA annotation needs manual editing, and additionally the identification of the IR region was not supported by this tool. CpGAVAS results showed high similarity with the GeSeq findings. If a cp genome belonging to a closely related taxon is available, the GeSeq annotation tool is the most useful for the analysis. In other cases, annotation with both GeSeq and cpGAVAS, followed by comparison of the results from both tools, provides the most precise information about functional genes and locations with minimal configuration.

Highly conserved structure, similar gene content and order were determined by comparative genome analysis of cv Tombul genome with seven different species, indicating that cv Tombul cp genome contains largely the same coding genes, tRNAs and rRNAs. However, the length of the cp genome from cv Tombul slightly differed from the published sequence KX822768 in GenBank, from which it could be inferred that some genetic differences exist even between cultivars.

### Repeat analysis

Simple sequence repeats (SSR) are useful for characterization of genetic diversity and development of molecular markers for phylogenetic studies and breeding. Herein, we identified several microsatellite sequences in the *C. avellana* cv Tombul cp genome, most of which were distributed in the intergenic regions, although some SSRs were detected in coding genes. Multiple SSR types were detected in the *ycf1* protein-coding gene, confirming the results of comparative genome analysis that *ycf1* contains high variability regions. The majority of simple repeats from the cv Tombul cp genome were classified as mononucleotides and dinucleotides, which were mainly composed of adenine (A) or thymine (T) repeats, and rarely contained guanine (G) or cytosine (C). Our findings presented similar results to previous studies [7, 63, 64]. These features could provide deeper information for phylogenetic research of *Corylus* by allowing development of species- and variety-specific molecular markers.

### Phylogenetic analysis

Previous studies have resolved the relationships of Betulaceae family, and most taxonomists have agreed that the Betulaceae family is divided into two subfamilies, named as Betuloideae (genera: *Alnus* and *Betula*) and Coryloideae (genera: *Corylus*, *Carpinus*, *Ostrya*, and *Ostryopsis*) [51]. The generic relationships within Coryloideae were studied by molecular markers including *matK* and *rbcL* genes, and ITS regions [51, 65–67]. We found a similar grouping within the cp genome phylogeny, including that *Corylus* formed a monophyletic group and had a close relationship with *Carpinus* and *Ostrya*: the results of our analyses supported the previous studies [52]. *Ostrya* formed a sister group with *Carpinus*, and these genera together constituted a sister group to *Corylus*. Whereas relationships between sub-families have been fairly well resolved, the inter-specific relationships within *Corylus* have not been completely determined due to lack of information about taxon sampling for *Corylus* species, and limited studies at the molecular level [29]. Our results about the relationship of *Corylus* species supported those recently reported by Yang et al. [7]; *Corylus avellana* cv Tombul exhibited a close relationship to the sister group of *C. fargesii* and *C. chinensis*, which are tree species in *Corylus* family, and the most distant relationship to the primitive species, *C. wangii*. As briefly stated, the complete cp genomes provide more in-depth information about both inter- and intraspecific relationships, and for evolutionary studies.

## Conclusion

*Corylus* is a phylogenetically and economically important genus with 16–20 species, in the family Betulaceae. Because of commercial and ornamental values of *Corylus* species, greater study of this genus can contribute to both science and the economy. In this study, we assembled the *C. avellana* cv Tombul cp genome by using WGS sequences generated as part of a whole genome sequencing project. The cp genome of cv Tombul has a typical cp genome structure, and is highly similar to other cp genomes of the *Betulaceae* family. Our results confirm that complete and highly-accurate chloroplast genome assemblies can be simply obtained from next generation whole genome shotgun data, but that assembly and annotation tools must be carefully selected and cross-checked for potential errors. Although the results were similar in all annotation tools in terms of gene content, GeSeq and cpGAVAS provided better results for gene locations. If the location information of exons and introns of a gene was needed for further analysis, annotation should be carried by using GeSeq. According to phylogenetic analysis, it was observed that *Corylus avellana* cv Tombul had a close relationship to *C. fargesii* and *C. chinensis*. Therefore, these two *Corylus* species may be especially useful for crossbreeding and grafting with *C. avellana*, in order to produce resilient and productive varieties. Moreover, the interspecific relationships of genus *Corylus* could be more precisely understood with enhanced taxon sampling. In the future, we are considering wider cp genome sampling of other cultivated varieties, to investigate whether cultivar specific markers exist or not, and the development of molecular markers for deeper information about phylogeny.

## Methods

### DNA extraction and sequencing

DNA extraction and sequencing was carried out as part of an ongoing *C. avellana* genome sequencing project. High molecular weight DNA was extracted from young leaf buds using a CTAB method optimized for Betulaceae [68]. Whole genome shotgun libraries were prepared using TruSeq kits and selected for an insert size of 600–800 nt. Paired-end sequencing was carried out on a Illumina HiSeq4000 and reads were deposited in the European Nucleotide Archive (Project accession: PRJEB31933). NanoPore sequencing reads were also obtained for the same cv Tombul genome project. NanoPore sequencing was carried out on the MinION platform using R9.4 flowcells and Ligation Sequencing Kit 1D, according to the manufacturer’s protocols (Oxford NanoPore Technologies, Oxford, UK).

### Chloroplast genome assembly and annotation

Whole genome Illumina paired-end raw data without adapters were used in de novo assembler NOVOPlasty, a seed-extend based assembler [37] (Fig. 2). The cp genome was assembled from WGS data, initiated by a seed sequence, which is iteratively extended bidirectionally, to obtain the circular genome. Using a reference genome is optional in the pipeline, but can be useful to obtain a single circular genome, and to eliminate manual adjustments. In this study, *Arabidopsis thaliana* (KX551970.1) and *Corylus avellana* complete cpDNA sequences (KX822768.2) were used as seed and reference genomes, respectively. We specified the following parameters: automatic insert size detection, a genome size range from 120,000 to 200,000, a K-mer value of 39, an insert range of 1.8, a strict insert range of 1.3, and the paired-end reads option. Moreover, the contig was checked using BLAST searches against the available complete cp sequence of KX822768 [47]. Relative positions were manually curated according to the reference genome, and the complete cp genome for Tombul cultivar was finally acquired for further analysis. In addition, Illumina paired-end raw sequence reads were processed by Trimmomatic to remove adapters, and trimmed sequences were assembled using ABySS 1.9 [39, 69]. Then, the cv Tombul cp genome obtained from NOVOplasty was aligned to the ABySS contigs using BLAST.

The Tombul cp genome was annotated through three different online programs, GeSeq, CpGAVAS and DOGMA with default parameters [40–43]. For the annotation file, the gene locations were compared and accepted when they matched the same position with at least two annotation tools. MEGA pairwise alignment was additionally used to confirm the genes among closely related taxa, and the gene locations were verified from cv Tombul cp genome sequences. Protein-coding and tRNA genes found by only one tool were not included in the map. The visual image of annotation was illustrated with the help of OGDRAW [70]. The final assembly was submitted to GenBank (MN082371).

### Comparative chloroplast genomic analysis

Complete cp genomes of seven species, including *Corylus avellana* (GenBank accession number: KX822768.2), *Betula nana* (GenBank accession number: NC_033978.1), *Juglans regia* (GenBank accession number: MF167463.1), *Populus trichocarpa* (GenBank accession number: EF489041.1), *Quercus rubra* (GenBank accession number: JX970937.1), *Glycine max* (GenBank accession number: NC_007942.1) and *Arabidopsis thaliana* (GenBank accession number: KX551970.1), were downloaded from NCBI, in order to compare the overall similarities among different cp genomes with Tombul cultivar. Pairwise alignments were implemented in the LAGAN alignment program included in mVISTA program with default parameters [48] using the annotation of *Corylus avellana* cv Tombul (Betulaceae, Fagales; GenBank accession number: MN082371) as the reference.

### SSR analysis

Simple sequence repeats (SSRs) were detected using two different microsatellite identification web tools, MISA (MIcroSAtellite identification tool) and IMEx-web (Imperfect Microsatellite Extraction Webserver) by setting the minimum number of repeats to 10, 5, 4, 3, 3 and 3 for mono-, di-, tri-, tetra-, penta- and hexanucleotides, respectively [49, 50].

### Phylogenomic analysis

The complete cp genome sequences of 22 species from Fagales were used for phylogenetic analysis, including representatives of genera from the Betulaceae, Fagaceae, and Juglandaceae. The cp genomes of species were aligned with multiple sequence alignment tool, MUSCLE [71]. All sequence gaps were excluded after alignment in the analysis. The evolutionary history was inferred by using the Maximum Likelihood method and Tamura-Nei model and analyses were conducted in MEGA X [72, 73]. The bootstrap consensus tree inferred from 500 replicates was taken to represent the evolutionary history of the taxa analyzed. All positions with less than 90% site coverage were eliminated, i.e., fewer than 10% alignment gaps, missing data, and ambiguous bases were allowed at any position (partial deletion option).

## Supplementary information

**Additional file 1: Table S1.** Comparison of three different annotation tools in terms of protein-coding gene content. **Table S2.** Comparison of three different annotation tools in terms of transfer and ribosomal RNA gene content. **Table S3.** Nucleotide changes in protein-coding genes. **Table S4.** BLAST result of the cv Tombul chloroplast genome against Viridiplantae (best 100 hits). **Table S5.** Simple sequence repeats within the cv Tombul chloroplast genome. **Table S6.** Comparison of two SSR identification tools for the cv Tombul chloroplast genome. **Table S7.** Differences between cv Tombul and KX822768 cp genome published in GenBank. **Table S8.** The features of Fagales and Malpighiales plastomes. **Table S9.** Differences between annotation tools. **Figure S1.** Number of classified SSR repeat types (considering complementary sequences). **Figure S2.** Schematic that explains the structure of cv Tombul chloroplast genome.

## Data Availability

The raw sequencing data used in this study are available in the ENA repository (project accession: PRJEB31933; https://www.ebi.ac.uk/ena/data/view/PRJEB31933). The full *C. avellana* cv. Tombul chloroplast assembly and annotation is available in the Genbank repository (Accession no: MN082371; https://www.ncbi.nlm.nih.gov/nuccore/MN082371).

## Abbreviations

A: Adenine
AFLP: Amplified fragment length polymorphism
BS: Bootstrap
C: Cytosine
cp: Chloroplast
cpDNA: Chloroplast DNA
cv: Cultivar
G: Guanine
IR: Inverted repeat
ISSR: Inter simple sequence repeats
ITS: Internal transcribed spacer
LSC: Large single copy
RAPD: Random amplified polymorphic DNA
SSC: Small single copy
SSR: Simple sequence repeat
T: Thymine
WGS: Whole genome shotgun
