# Supplementary material for: Comparison of different annotation tools for characterization of the complete chloroplast genome of Corylus avellana cv Tombul
Source: BMC Genomics. 2019 Nov 20;20:874. doi: 10.1186/s12864-019-6253-5 (PMC6865063; doi:10.1186/s12864-019-6253-5)
Supplement: Supplementary file 1 — Additional file 1: Table S1. Comparison of three different annotation tools in terms of protein-coding gene content. Table S2. Comparison of three different annotation tools in terms of transfer and ribosomal RNA gene content. Table S3. Nucleotide changes in protein-coding genes. Table S4. BLAST result of the cv Tombul chloroplast genome against Viridiplantae (best 100 hits). Table S5. Simple sequence repeats within the cv Tombul chloroplast genome. Table S6. Comparison of two SSR identification tools for the cv Tombul chloroplast genome. Table S7. Differences between cv Tombul and KX822768 cp genome published in GenBank. Table S8. The features of Fagales and Malpighiales plastomes. Table S9. Differences between annotation tools. Figure S1. Number of classified SSR repeat types (considering complementary sequences). Figure S2. Schematic that explains the structure of cv Tombul chloroplast genome. [file 12864_2019_6253_MOESM1_ESM.docx]

**Table S1** Comparison of three different annotation tools in terms of protein-coding gene content

| cpGAVAS | | | |  | Dogma | | | |  | GeSeq | | | |
| --- | --- | --- | --- | --- | --- | --- | --- | --- | --- | --- | --- | --- | --- |
| Genes | **Start** | **End** | **Strand** |  | **Genes** | **Start** | **End** | **Strand** |  | **Genes** | **Start** | **End** | **Strand** |
| *accD* | 61672 | 63231 | + |  | *accD* | 61672 | 63207 | + |  | *accD* | 61709 | 63205 | + |
| *atpA* | 11893 | 13416 | - |  | *atpA* | 11899 | 13416 | - |  | *atpA* | 11891 | 13417 | - |
| *atpB* | 57234 | 58735 | - |  | *atpB* | 57266 | 58735 | - |  | *atpB* | 57234 | 58735 | - |
| *atpE* | 56839 | 57237 | - |  | *atpE* | 56842 | 57237 | - |  | *atpE* | 56841 | 57237 | - |
| *atpF* | 13485 | 14789 | - |  | *atpF* | 13488 | 13955 | - |  | *atpF* | 13486 | 13895 | - |
|  |  |  |  |  | *atpF* | 14631 | 14789 | - |  | *atpF* | 14645 | 14789 | - |
| *atpH* | 15314 | 15559 | - |  | *atpH* | 15317 | 15559 | - |  | *atpH* | 15314 | 15559 | - |
| *atpI* | 16779 | 17525 | - |  | *atpI* | 16782 | 17525 | - |  | *atpI* | 16780 | 17525 | - |
| *ccsA* | 121141 | 122097 | + |  | *ccsA* | 121141 | 122094 | + |  | *ccsA* | 121141 | 122088 | + |
| *cemA* | 65565 | 66254 | + |  | *cemA* | 65565 | 66251 | + |  | *cemA* | 65506 | 66254 | + |
| *clpP* | 76125 | 78145 | - |  | *clpP* | 76128 | 76352 | - |  | *clpP* | 76966 | 77264 | - |
|  |  |  |  |  | *clpP* | 76966 | 77259 | - |  | *clpP* | 76125 | 76352 | - |
|  |  |  |  |  | *clpP* | 78077 | 78145 | - |  | *clpP* | 78074 | 78145 | - |
| *infA* | 85877 | 86110 | - |  | *infA* | 85880 | 86110 | - |  | *infA* | 85878 | 86106 | - |
| *matK* | 2172 | 3686 | - |  | *matK* | 2175 | 3686 | - |  | *matK* | 2172 | 3686 | - |
| *ndhA* | 126653 | 128887 | - |  | *ndhA* | 126656 | 127192 | - |  | *ndhA* | 126654 | 127193 | - |
|  |  |  |  |  | *ndhA* | 128336 | 128887 | - |  | *ndhA* | 128320 | 128887 | - |
| *ndhB* | 100825 | 103049 | - |  | *ndhB* | 100828 | 101586 | - |  | *ndhB* | 100826 | 101586 | - |
| *ndhB* | 148816 | 151040 | + |  | *ndhB* | 102273 | 103049 | - |  | *ndhB* | 102272 | 103049 | - |
|  |  |  |  |  | *ndhB* | 148816 | 149592 | + |  | *ndhB* | 148816 | 149593 | + |
|  |  |  |  |  | *ndhB* | 150279 | 151037 | + |  | *ndhB* | 150279 | 151039 | + |
|  |  |  |  |  |  |  |  |  |  | *ndhB* | 117887 | 118194 | - |
| *ndhC* | 54736 | 55098 | - |  | *ndhC* | 54739 | 55098 | - |  | *ndhC* | 54737 | 55098 | - |
| *ndhD* | 122428 | 123921 | - |  | *ndhD* | 122425 | 123924 | - |  | *ndhD* | 122425 | 123924 | - |
|  |  |  |  |  |  |  |  |  |  | *ndhD* | 117870 | 118170 | - |
| *ndhE* | 124586 | 124891 | - |  | *ndhE* | 124589 | 124891 | - |  | *ndhE* | 124587 | 124891 | - |
| *ndhF* | 116655 | 118883 | - |  | *ndhF* | 116658 | 118883 | - |  | *ndhF* | 116659 | 118883 | - |
|  |  |  |  |  |  |  |  |  |  | *ndhF* | 122910 | 123269 | - |
| *ndhG* | 125123 | 125653 | - |  | *ndhG* | 125126 | 125653 | - |  | *ndhG* | 125121 | 125653 | - |
| *ndhH* | 128889 | 130070 | - |  | *ndhH* | 128892 | 130070 | - |  | *ndhH* | 128889 | 130069 | - |
| *ndhI* | 126079 | 126570 | - |  | *ndhI* | 126076 | 126570 | - |  | *ndhI* | 126075 | 126570 | - |
| *ndhJ* | 53367 | 53843 | - |  | *ndhJ* | 53370 | 53843 | - |  | *ndhJ* | 53368 | 53842 | - |
| *ndhK* | 53998 | 54856 | - |  | *ndhK* | 54001 | 54714 | - |  | *ndhK* | 53999 | 54677 | - |
| *ndhK* | 53998 | 54678 | - |  | *ndhK* | 54692 | 54856 | - |  |  |  |  |  |
|  |  |  |  |  |  |  |  |  |  | *pbf1* | 80504 | 80635 | - |
| *petA* | 66468 | 67434 | + |  | *petA* | 66468 | 67139 | + |  | *petA* | 66468 | 67434 | + |
|  |  |  |  |  | *petA* | 67114 | 67431 | + |  |  |  |  |  |
| *petB* | 81835 | 82538 | + |  | *petB* | 81885 | 82535 | + |  | *petB* | 81895 | 82537 | + |
| *petD* | 83326 | 83871 | + |  | *petD* | 83326 | 83868 | + |  | *petD* | 83375 | 83849 | + |
| *petG* | 72234 | 72347 | + |  | *petG* | 72234 | 72344 | + |  | *petG* | 72234 | 72348 | + |
| *petL* | 71964 | 72059 | + |  | *petL* | 71964 | 72056 | + |  | *petL* | 71964 | 72059 | + |
| *petN* | 31354 | 31452 | + |  | *petN* | 31357 | 31449 | + |  | *petN* | 31363 | 31452 | + |
| *psaA* | 43785 | 46037 | - |  | *psaA* | 43788 | 46037 | - |  | *psaA* | 43786 | 46037 | - |
|  |  |  |  |  |  |  |  |  |  | *psaA* | 41712 | 43727 | - |
| *psaB* | 41555 | 43759 | - |  | *psaB* | 41558 | 43759 | - |  | *psaB* | 41556 | 43759 | - |
|  |  |  |  |  |  |  |  |  |  | *psaB* | 43815 | 45914 | - |
| *psaC* | 124066 | 124311 | - |  | *psaC* | 124069 | 124311 | - |  | *psaC* | 124066 | 124311 | - |
| *psaI* | 64061 | 64174 | + |  | *psaI* | 64061 | 64171 | + |  | *psaI* | 64061 | 64173 | + |
| *psaJ* | 73140 | 73274 | + |  | *psaJ* | 73140 | 73271 | + |  | *psaJ* | 73140 | 73269 | + |
|  |  |  |  |  |  |  |  |  |  | *psb30* | 45897 | 45938 | + |
| *psbA* | 530 | 1591 | - |  | *psbA* | 533 | 1591 | - |  | *psbA* | 530 | 1591 | - |
|  |  |  |  |  |  |  |  |  |  | *psbA* | 162197 | 163258 | - |
|  |  |  |  |  |  |  |  |  |  | *psbA* | 37213 | 37777 | + |
| *psbB* | 78611 | 80137 | + |  | *psbB* | 78611 | 80131 | + |  | *psbB* | 78611 | 80137 | + |
| *psbC* | 37856 | 39319 | + |  | *psbC* | 37856 | 39316 | + |  | *psbC* | 37934 | 39318 | + |
| *psbD* | 36889 | 37950 | + |  | *psbD* | 36889 | 37947 | + |  | *psbD* | 36889 | 37950 | + |
|  |  |  |  |  |  |  |  |  |  | *psbD* | 698 | 1247 | - |
|  |  |  |  |  |  |  |  |  |  | *psbD* | 162365 | 162914 | - |
| *psbE* | 70641 | 70892 | - |  | *psbE* | 70644 | 70892 | - |  | *psbE* | 70641 | 70892 | - |
|  |  |  |  |  |  |  |  |  |  | *psbE* | 26478 | 26593 | - |
| *psbF* | 68982 | 69101 | - |  | *psbF* | 68985 | 69101 | - |  | *psbF* | 68982 | 69101 | - |
| *psbF* | 70512 | 70631 | - |  | *psbF* | 70515 | 70631 | - |  | *psbF* | 70512 | 70631 | - |
| *psbH* | 80742 | 80972 | + |  | *psbH* | 80751 | 80969 | + |  | *psbH* | 80751 | 80972 | + |
| *psbI* | 9175 | 9330 | + |  | *psbI* | 9175 | 9327 | + |  | *psbI* | 9220 | 9330 | + |
| *psbJ* | 68581 | 68703 | - |  | *psbJ* | 68584 | 68703 | - |  | *psbJ* | 68582 | 68703 | - |
| *psbJ* | 70111 | 70233 | - |  | *psbJ* | 70114 | 70233 | - |  | *psbJ* | 70112 | 70233 | - |
| *psbK* | 8617 | 8808 | + |  | *psbK* | 8623 | 8805 | + |  | *psbK* | 8623 | 8808 | + |
| *psbL* | 68843 | 68959 | - |  | *psbL* | 68846 | 68959 | - |  | *psbL* | 68844 | 68959 | - |
| *psbL* | 70373 | 70489 | - |  | *psbL* | 70376 | 70489 | - |  | *psbL* | 70374 | 70489 | - |
| *psbM* | 32408 | 32521 | - |  | *psbM* | 32420 | 32521 | - |  | *psbM* | 32418 | 32520 | - |
| *psbN* | 80504 | 80635 | - |  | *psbN* | 80507 | 80635 | - |  |  |  |  |  |
| *psbT* | 80315 | 80431 | + |  | *psbT* | 80315 | 80428 | + |  | *psbT* | 80324 | 80428 | + |
| *psbZ* | 40087 | 40275 | + |  | *psbZ* | 40087 | 40272 | + |  | *psbZ* | 40087 | 40275 | + |
| *rbcL* | 59556 | 60983 | + |  | *rbcL* | 59556 | 60980 | + |  | *rbcL* | 59556 | 60982 | + |
| *rpl2* | 90542 | 92054 | - |  | *rpl2* | 90545 | 91012 | - |  | *rpl2* | 90544 | 90981 | - |
| *rpl2* | 159811 | 161323 | + |  | *rpl2* | 91662 | 92054 | - |  | *rpl2* | 91664 | 92054 | - |
|  |  |  |  |  | *rpl2* | 159811 | 160203 | + |  | *rpl2* | 159811 | 160201 | + |
|  |  |  |  |  | *rpl2* | 160853 | 161320 | + |  | *rpl2* | 160884 | 161321 | + |
| *rpl14* | 86816 | 87184 | - |  | *rpl14* | 86819 | 87184 | - |  | *rpl14* | 86817 | 87184 | - |
| *rpl16* | 87328 | 87738 | - |  | *rpl16* | 87331 | 87729 | - |  | *rpl16* | 87331 | 87729 | - |
| *rpl20* | 74693 | 75046 | - |  | *rpl20* | 74696 | 75046 | - |  | *rpl20* | 74694 | 75046 | - |
| *rpl22* | 89622 | 90131 | - |  | *rpl22* | 89625 | 90131 | - |  | *rpl22* | 89757 | 90126 | - |
| *rpl23* | 92073 | 92354 | - |  | *rpl23* | 92076 | 92354 | - |  | *rpl23* | 92075 | 92354 | - |
| *rpl23* | 159511 | 159792 | + |  | *rpl23* | 159511 | 159789 | + |  | *rpl23* | 159511 | 159790 | + |
| *rpl32* | 119796 | 119959 | - |  | *rpl32* | 119796 | 119945 | + |  | *rpl32* | 119796 | 119937 | + |
| *rpl33* | 73754 | 73975 | + |  | *rpl33* | 73754 | 73972 | + |  | *rpl33* | 73754 | 73974 | + |
| *rpl36* | 85643 | 85756 | + |  | *rpl36* | 85646 | 85756 | - |  | *rpl36* | 85643 | 85756 | - |
| *rpoA* | 84019 | 85014 | - |  | *rpoA* | 84040 | 85014 | - |  | *rpoA* | 84024 | 85014 | - |
| *rpoB* | 25934 | 29146 | - |  | *rpoB* | 25937 | 29146 | - |  | *rpoB* | 25935 | 29146 | - |
| *rpoC1* | 23021 | 25928 | - |  | *rpoC1* | 23024 | 24646 | - |  | *rpoC1* | 23022 | 24641 | - |
|  |  |  |  |  | *rpoC1* | 25467 | 25907 | - |  | *rpoC1* | 25467 | 25907 | - |
| *rpoC2* | 18671 | 22855 | - |  | *rpoC2* | 18674 | 22855 | - |  | *rpoC2* | 18674 | 22843 | - |
| *rps2* | 17746 | 18450 | - |  | *rps2* | 17749 | 18450 | - |  | *rps2* | 17748 | 18450 | - |
| *rps3* | 88981 | 89637 | - |  | *rps3* | 88984 | 89637 | - |  | *rps3* | 88984 | 89637 | - |
| *rps4* | 49624 | 50229 | - |  | *rps4* | 49627 | 50229 | - |  | *rps4* | 49625 | 50229 | - |
| *rps7* | 103369 | 103836 | - |  | *rps7* | 103372 | 103836 | - |  | *rps7* | 103370 | 103836 | - |
| *rps7* | 148029 | 148496 | + |  | *rps7* | 148029 | 148493 | + |  | *rps7* | 148029 | 148495 | + |
| *rps8* | 86237 | 86641 | - |  | *rps8* | 86240 | 86641 | - |  | *rps8* | 86237 | 86641 | - |
| *rps11* | 85084 | 85500 | - |  | *rps11* | 85087 | 85500 | - |  | *rps11* | 85085 | 85500 | - |
| *rps12* | 75840 | 75986 | - |  | *rps12* | 75873 | 75986 | - |  | *rps12* | 75872 | 75986 | - |
| *rps12* | 104447 | 104755 | - |  | *rps12* | 104450 | 104689 | - |  | *rps12* | 103892 | 103921 | - |
| *rps12* | 147110 | 147418 | + |  | *rps12* | 147176 | 147415 | + |  | *rps12* | 104456 | 104690 | - |
|  |  |  |  |  | *rps12_3end* | 104438 | 104689 | - |  | *rps12* | 147175 | 147409 | + |
| *rps14* | 41122 | 41424 | - |  | *rps14* | 41125 | 41424 | - |  | *rps14* | 41123 | 41424 | - |
| *rps15* | 130193 | 130465 | - |  | *rps15* | 130196 | 130492 | - |  | *rps15* | 130194 | 130465 | - |
| *rps16* | 5540 | 5773 | - |  | *rps16* | 5570 | 5773 | - |  | *rps16* | 5559 | 5773 | - |
|  |  |  |  |  |  |  |  |  |  | *rps16* | 6643 | 6690 | - |
|  |  |  |  |  |  |  |  |  |  | *rps16* | 167226 | 167440 | - |
|  |  |  |  |  |  |  |  |  |  | *rps16* | 168310 | 168357 | - |
| *rps18* | 74117 | 74474 | + |  | *rps18* | 74117 | 74419 | + |  | *rps18* | 74118 | 74418 | + |
| *rps19* | 90198 | 90476 | - |  | *rps19* | 90201 | 90476 | - |  | *rps19* | 90199 | 90476 | - |
|  |  |  |  |  | *rps19* | 161389 | 161664 | + |  | *rps19* | 161389 | 161666 | + |
| *ycf1* | 131190 | 136511 | - |  | *ycf1* | 130791 | 136511 | - |  | *ycf1* | 130784 | 136511 | - |
|  |  |  |  |  | *ycf1* | 115354 | 116562 | + |  | *ycf1* | 115354 | 116563 | + |
| *ycf2* | 93513 | 99503 | + |  | *ycf2* | 92682 | 99500 | + |  | *ycf2* | 92682 | 99505 | + |
| *ycf2* | 152362 | 158352 | - |  | *ycf2* | 152365 | 159183 | - |  | *ycf2* | 152360 | 159183 | - |
| *ycf3* | 46825 | 48817 | - |  | *ycf3* | 46828 | 46977 | - |  | *ycf3* | 46827 | 46982 | - |
|  |  |  |  |  | *ycf3* | 47753 | 47980 | - |  | *ycf3* | 47753 | 47982 | - |
|  |  |  |  |  | *ycf3* | 48692 | 48817 | - |  | *ycf3* | 48691 | 48817 | - |
| *ycf4* | 64588 | 65142 | + |  | *ycf4* | 64588 | 65139 | + |  | *ycf4* | 64589 | 65142 | + |
| *ycf15* | 99594 | 100135 | + |  | *ycf15* | 99645 | 99776 | + |  |  |  |  |  |
| *ycf15* | 105501 | 105677 | - |  | *ycf15* | 99752 | 99874 | + |  |  |  |  |  |
| *ycf15* | 146188 | 146364 | + |  | *ycf15* | 151991 | 152113 | - |  |  |  |  |  |
| *ycf15* | 151988 | 152220 | - |  | *ycf15* | 152089 | 152220 | - |  |  |  |  |  |
|  |  |  |  |  | *ycf68* | 108683 | 108991 | + |  |  |  |  |  |
|  |  |  |  |  | *ycf68* | 142874 | 143182 | - |  |  |  |  |  |

**Table S2** Comparison of three different annotation tools in terms of transfer and ribosomal RNA gene content

| cpGAVAS | | | |  | Dogma | | | |  | GeSeq | | | |
| --- | --- | --- | --- | --- | --- | --- | --- | --- | --- | --- | --- | --- | --- |
| tRNA | **Start** | **End** | **Strand** |  | **tRNA** | **Start** | **End** | **Strand** |  | **tRNA** | **Start** | **End** | **Strand** |
|  |  |  |  |  | *trnA-TGC* | 109564 | 109601 | + |  |  |  |  |  |
|  |  |  |  |  | *trnA-TGC* | 110411 | 110445 | + |  |  |  |  |  |
|  |  |  |  |  | *trnA-TGC* | 141420 | 141454 | - |  |  |  |  |  |
|  |  |  |  |  | *trnA-TGC* | 142264 | 142301 | - |  |  |  |  |  |
| *trnC-GCA* | 30414 | 30484 | + |  | *trnC-GCA* | 30414 | 30493 | + |  | *trnC-GCA* | 30414 | 30484 | + |
| *trnD-GTC* | 33706 | 33779 | - |  | *trnD-GTC* | 33706 | 33779 | - |  | *trnD-GTC* | 33706 | 33779 | - |
| *trnE-TTC* | 34408 | 34480 | - |  | *trnE-TTC* | 34408 | 34480 | - |  | *trnE-TTC* | 34408 | 34480 | - |
| *trnF-GAA* | 52451 | 52523 | + |  | *trnF-GAA* | 52451 | 52523 | + |  | *trnF-GAA* | 52451 | 52523 | + |
| *trnfM-CAT* | 40885 | 40958 | - |  | *trnfM-CAT* | 40885 | 40958 | - |  |  |  |  |  |
| *trnG-GCC* | 40604 | 40674 | + |  | *trnG-GCC* | 40604 | 40674 | + |  | *trnG-GCC* | 40604 | 40674 | + |
|  |  |  |  |  | *trnG-TCC* | 11207 | 11243 | + |  |  |  |  |  |
| *trnH-GTG* | 17 | 90 | - |  | *trnH-GTG* | 17 | 91 | - |  | *trnH-GTG* | 17 | 90 | - |
|  |  |  |  |  |  |  |  |  |  | *trnH-GTG* | 161684 | 161757 | - |
| *trnI-CAT* | 92520 | 92593 | - |  | *trnI-CAT* | 92520 | 92593 | - |  |  |  |  |  |
| *trnI-CAT* | 159272 | 159345 | + |  | *trnI-CAT* | 159272 | 159345 | + |  |  |  |  |  |
|  |  |  |  |  | *trnI-GAT* | 108472 | 108513 | + |  | *trnI-GAT* | 108472 | 108559 | + |
|  |  |  |  |  | *trnI-GAT* | 109465 | 109499 | + |  | *trnI-GAT* | 143306 | 143393 | - |
|  |  |  |  |  | *trnI-GAT* | 142366 | 142400 | - |  |  |  |  |  |
|  |  |  |  |  | *trnI-GAT* | 143352 | 143393 | - |  |  |  |  |  |
|  |  |  |  |  | *trnK-TTT* | 4442 | 4478 | - |  |  |  |  |  |
| *trnL-CAA* | 100167 | 100247 | - |  | *trnL-CAA* | 100167 | 100247 | - |  | *trnL-CAA* | 100167 | 100247 | - |
| *trnL-CAA* | 151618 | 151698 | + |  | *trnL-CAA* | 151618 | 151698 | + |  | *trnL-CAA* | 151618 | 151698 | + |
| *trnL-TAG* | 120950 | 121029 | + |  | *trnL-TAG* | 120950 | 121029 | + |  | *trnL-TAG* | 120950 | 121029 | + |
|  |  |  |  |  | *trnL-TAA* | 51438 | 51474 | + |  |  |  |  |  |
|  |  |  |  |  | *trnL-TAA* | 52012 | 52061 | + |  |  |  |  |  |
| *trnM-CAT* | 56543 | 56615 | + |  | *trnM-CAT* | 56544 | 56615 | + |  | *trnM-CAT* | 56543 | 56615 | + |
|  |  |  |  |  | *trnM-CAT* | 35416 | 35474 | + |  | *trnM-CAT* | 40885 | 40958 | - |
|  |  |  |  |  |  |  |  |  |  | *trnM-CAT* | 92520 | 92593 | - |
|  |  |  |  |  |  |  |  |  |  | *trnM-CAT* | 159272 | 159345 | + |
| *trnN-GTT* | 114958 | 115029 | - |  | *trnN-GTT* | 114958 | 115029 | - |  | *trnN-GTT* | 136836 | 136907 | + |
| *trnN-GTT* | 136836 | 136907 | + |  | *trnN-GTT* | 136836 | 136907 | + |  | *trnN-GTT* | 114958 | 115029 | - |
| *trnP-TGG* | 72661 | 72734 | - |  | *trnP-TGG* | 72661 | 72734 | - |  | *trnP-TGG* | 72661 | 72734 | - |
|  |  |  |  |  | *trnP-GGG* | 72663 | 72733 | - |  |  |  |  |  |
| *trnQ-TTG* | 8107 | 8178 | - |  | *trnQ-TTG* | 8107 | 8178 | - |  | *trnQ-TTG* | 8107 | 8178 | - |
|  |  |  |  |  |  |  |  |  |  | *trnQ-TTG* | 169774 | 169845 | - |
| *trnR-ACG* | 114255 | 114328 | + |  | *trnR-ACG* | 114255 | 114328 | + |  | *trnR-ACG* | 114255 | 114328 | + |
| *trnR-ACG* | 137537 | 137610 | - |  | *trnR-ACG* | 137537 | 137610 | - |  | *trnR-ACG* | 137537 | 137610 | - |
| *trnR-TCT* | 11552 | 11623 | + |  | *trnR-TCT* | 11552 | 11623 | + |  | *trnR-TCT* | 11552 | 11623 | + |
| *trnS-GCT* | 9479 | 9566 | - |  | *trnS-GCT* | 9479 | 9566 | - |  | *trnS-GCT* | 9479 | 9566 | - |
| *trnS-TGA* | 39570 | 39662 | - |  | *trnS-TGA* | 39570 | 39662 | - |  | *trnS-TGA* | 39570 | 39662 | - |
| *trnS-GGA* | 49229 | 49315 | + |  | *trnS-GGA* | 49229 | 49315 | + |  | *trnS-GGA* | 49229 | 49315 | + |
|  |  |  |  |  |  |  |  |  |  | *trnS-GCT* | 171146 | 171233 | - |
| *trnT-GGT* | 35410 | 35481 | + |  | *trnT-GGT* | 35410 | 35481 | + |  | *trnT-GGT* | 35410 | 35481 | + |
| *trnT-TGT* | 50563 | 50635 | - |  | *trnT-TGT* | 50563 | 50635 | - |  | *trnT-TGT* | 50563 | 50635 | - |
|  |  |  |  |  | *trnT-GGT* | 56549 | 56607 | + |  |  |  |  |  |
| *trnV-GAC* | 106388 | 106459 | + |  | *trnV-GAC* | 106388 | 106459 | + |  | *trnV-GAC* | 106388 | 106459 | + |
| *trnV-GAC* | 145406 | 145477 | - |  | *trnV-GAC* | 145406 | 145477 | - |  | *trnV-GAC* | 145406 | 145477 | - |
|  |  |  |  |  | *trnV-TAC* | 55655 | 55690 | - |  |  |  |  |  |
|  |  |  |  |  | *trnV-TAC* | 56332 | 56369 | - |  |  |  |  |  |
| *trnW-CCA* | 72423 | 72496 | - |  | *trnW-CCA* | 72423 | 72496 | - |  | *trnW-CCA* | 72423 | 72496 | - |
| *trnY-GTA* | 34264 | 34347 | - |  | *trnY-GTA* | 34264 | 34347 | - |  | *trnY-GTA* | 34264 | 34347 | - |
|  |  |  |  |  |  |  |  |  |  |  |  |  |  |
| *rrn4.5S* | 113510 | 113780 | + |  | *rrn4.5* | 113510 | 113612 | + |  | *rrn4.5* | 113511 | 113612 | + |
| *rrn4.5S* | 138085 | 138355 | - |  | *rrn4.5* | 138253 | 138355 | - |  | *rrn4.5* | 138253 | 138354 | - |
| *rrn5S* | 113872 | 113993 | + |  | *rrn5* | 113873 | 113993 | + |  | *rrn5* | 113873 | 113993 | + |
| *rrn5S* | 137872 | 137993 | - |  | *rrn5* | 137872 | 137992 | - |  | *rrn5* | 137872 | 137992 | - |
| *rrn16S* | 106687 | 108177 | - |  | *rrn16* | 106687 | 108177 | + |  | *rrn16* | 106687 | 108177 | + |
| *rrn16S* | 143688 | 145178 | + |  | *rrn16* | 143688 | 145178 | - |  | *rrn16* | 143688 | 145178 | - |
| *rrn23S* | 109977 | 113410 | - |  | *rrn23* | 110603 | 113411 | + |  | *rrn23* | 110603 | 113411 | + |
| *rrn23S* | 138455 | 141888 | + |  | *rrn23* | 138454 | 141262 | - |  | *rrn23* | 138454 | 141262 | - |

**Table S3** Nucleotide changes in protein-coding genes

| Genes | | Nucleotide Change | | Codon Change | | Aminoacid Change |
| --- | --- | --- | --- | --- | --- | --- |
| *ndhD* | A > G | | AUA > AUG | | I > M | |
| *petN* |  |  |  |  |  |  |
| *psbH* |  |  |  |  |  |  |
| *ycf2* | U > G | | AUU > AUG | | I > M | |
| *rpl32* |  |  |  |  |  |  |
| *psbI* | C > G | | AUC > AUG | | I > M | |
| *psbT* |  |  |  |  |  |  |
| *rpl16* |  |  |  |  |  |  |
| *rps16* |  |  |  |  |  |  |
| *rpl36* | C > A | | CUG > AUG | | L > M | |
| *rps19* | G > A | | GUG > AUG | | V > M | |

**Table S4** BLAST result of the cv Tombul chloroplast genome against Viridiplantae (best 100 hits)

| Description | Max Score | Total Score | Query Cover | E value | Per. Ident | Accession |
| --- | --- | --- | --- | --- | --- | --- |
| *Corylus heterophylla* var. sutchuenensis chloroplast, complete genome | 1.07E+05 | 3.96E+05 | 100% | 0 | 99.88% | MF996573.1 |
| *Corylus avellana* chloroplast, complete genome | 1.07E+05 | 3.94E+05 | 99% | 0 | 99.84% | KX822768.2 |
| *Corylus fargesii* chloroplast, complete genome | 1.26E+05 | 3.95E+05 | 100% | 0 | 99.69% | KX822767.2 |
| *Corylus* *chinensis* chloroplast, complete genome | 1.26E+05 | 3.95E+05 | 100% | 0 | 99.69% | KX814336.2 |
| *Corylus* *mandshurica* chloroplast, complete genome | 1.26E+05 | 3.92E+05 | 99% | 0 | 99.60% | NC_039127.1 |
| *Corylus* *mandshurica* chloroplast, complete genome | 1.26E+05 | 3.93E+05 | 99% | 0 | 99.60% | MF375334.1 |
| *Corylus* *heterophylla* chloroplast, complete genome | 1.26E+05 | 3.94E+05 | 100% | 0 | 99.53% | KX822769.2 |
| *Corylus* *ferox* var. thibetica chloroplast, complete genome | 1.26E+05 | 3.92E+05 | 99% | 0 | 99.51% | NC_039128.1 |
| *Corylus ferox* var. thibetica chloroplast, complete genome | 1.26E+05 | 3.93E+05 | 99% | 0 | 99.51% | MF375335.1 |
| *Corylus yunnanensis* chloroplast, complete genome | 93982 | 3.89E+05 | 99% | 0 | 99.51% | NC_039129.1 |
| *Corylus yunnanensis* chloroplast, complete genome | 93982 | 3.90E+05 | 99% | 0 | 99.51% | MF375336.1 |
| *Corylus wangii* chloroplast, complete genome | 93882 | 3.89E+05 | 99% | 0 | 99.46% | NC_040995.1 |
| *Corylus wangii* chloroplast, complete genome | 93882 | 3.89E+05 | 99% | 0 | 99.46% | MH628454.1 |
| *Carpinus putoensis* chloroplast, complete genome | 61658 | 3.76E+05 | 98% | 0 | 99.31% | KX695124.1 |
| *Ostrya japonica* isolate Oja15 chloroplast, complete genome | 81131 | 3.49E+05 | 88% | 0 | 99.25% | MG662143.1 |
| *Ostrya japonica* isolate Oja18 chloroplast, complete genome | 81131 | 3.49E+05 | 88% | 0 | 99.25% | MG662142.1 |
| *Ostrya japonica* isolate Oja26 chloroplast, complete genome | 81126 | 3.49E+05 | 88% | 0 | 99.25% | MG662137.1 |
| *Ostrya japonica* chloroplast, complete genome | 81109 | 3.40E+05 | 85% | 0 | 99.24% | MG386375.1 |
| *Ostrya japonica* isolate Oja01 chloroplast, complete genome | 81107 | 3.49E+05 | 88% | 0 | 99.24% | MG662148.1 |
| *Ostrya chinensis* isolate Och13 chloroplast, complete genome | 81091 | 3.49E+05 | 88% | 0 | 99.23% | MG662150.1 |
| *Ostrya japonica* isolate Oja02 chloroplast, complete genome | 81091 | 3.49E+05 | 88% | 0 | 99.23% | MG662147.1 |
| *Ostrya japonica* isolate Oja19 chloroplast, complete genome | 81091 | 3.49E+05 | 88% | 0 | 99.23% | MG662141.1 |
| *Ostrya rehderiana* isolate Ore01 chloroplast, complete genome | 81091 | 3.49E+05 | 88% | 0 | 99.23% | MG662135.1 |
| *Ostrya rehderiana* isolate Ore03 chloroplast, complete genome | 81091 | 3.49E+05 | 88% | 0 | 99.23% | MG662133.1 |
| *Ostrya chinensis* isolate Och02 chloroplast, complete genome | 81085 | 3.49E+05 | 88% | 0 | 99.23% | MG662154.1 |
| *Ostrya chinensis* isolate Och03 chloroplast, complete genome | 81085 | 3.49E+05 | 88% | 0 | 99.23% | MG662153.1 |
| *Ostrya chinensis* isolate Och12 chloroplast, complete genome | 81085 | 3.49E+05 | 88% | 0 | 99.23% | MG662151.1 |
| *Ostrya japonica* isolate Oja08 chloroplast, complete genome | 81085 | 3.49E+05 | 88% | 0 | 99.23% | MG662145.1 |
| *Ostrya japonica* isolate Oja20 chloroplast, complete genome | 81085 | 3.49E+05 | 88% | 0 | 99.23% | MG662140.1 |
| *Ostrya japonica* isolate Oja21 chloroplast, complete genome | 81085 | 3.49E+05 | 88% | 0 | 99.23% | MG662139.1 |
| *Ostrya rehderiana* isolate Ore02 chloroplast, complete genome | 81085 | 3.49E+05 | 88% | 0 | 99.23% | MG662134.1 |
| *Ostrya rehderiana* isolate Ore04 chloroplast, complete genome | 81085 | 3.49E+05 | 88% | 0 | 99.23% | MG662132.1 |
| *Ostrya japonica* isolate Oja06 chloroplast, complete genome | 81083 | 3.49E+05 | 88% | 0 | 99.23% | MG662146.1 |
| *Ostrya rehderiana* chloroplast, complete genome | 81078 | 3.71E+05 | 96% | 0 | 99.23% | KT454094.1 |
| *Ostrya japonica* isolate Oja23 chloroplast, complete genome | 81074 | 3.49E+05 | 88% | 0 | 99.23% | MG662138.1 |
| *Ostrya chinensis* isolate Och09 chloroplast, complete genome | 81030 | 3.44E+05 | 86% | 0 | 99.21% | MG662152.1 |
| *Ostrya chinensis* isolate Och08 chloroplast, complete genome | 80993 | 3.44E+05 | 86% | 0 | 99.19% | MG662156.1 |
| *Ostrya trichocarpa* isolate Otr07 chloroplast, complete genome | 80923 | 3.49E+05 | 88% | 0 | 99.18% | MG662128.1 |
| *Ostrya chinensis* isolate Och14 chloroplast, complete genome | 80965 | 3.44E+05 | 86% | 0 | 99.17% | NC_039817.1 |
| *Ostrya chinensis* isolate Och14 chloroplast, complete genome | 80965 | 3.44E+05 | 86% | 0 | 99.17% | MG662149.1 |
| *Ostrya japonica* isolate Oja27 chloroplast, complete genome | 85464 | 3.48E+05 | 88% | 0 | 99.16% | NC_039816.1 |
| *Ostrya japonica* isolate Oja27 chloroplast, complete genome | 85464 | 3.48E+05 | 88% | 0 | 99.16% | MG662136.1 |
| *Ostrya japonica* isolate Oja09 chloroplast, complete genome | 85452 | 3.48E+05 | 88% | 0 | 99.16% | MG662144.1 |
| *Ostrya japonica* isolate Oja10 chloroplast, complete genome | 85443 | 3.48E+05 | 88% | 0 | 99.16% | MG662157.1 |
| *Ostrya trichocarpa* isolate Otr04 chloroplast, complete genome | 80941 | 3.49E+05 | 88% | 0 | 99.16% | MG662129.1 |
| *Ostrya trichocarpa* isolate Otr03 chloroplast, complete genome | 80936 | 3.49E+05 | 88% | 0 | 99.16% | MG662130.1 |
| *Ostrya chinensis* isolate Och01 chloroplast, complete genome | 80926 | 3.49E+05 | 88% | 0 | 99.16% | MG662155.1 |
| *Carpinus betulus* chloroplast, complete genome | 85253 | 3.63E+05 | 92% | 0 | 99.12% | NC_039934.1 |
| *Carpinus betulus* chloroplast, complete genome | 85253 | 3.81E+05 | 99% | 0 | 99.12% | MF977767.1 |
| *Ostrya trichocarpa* isolate Otr01 chloroplast, complete genome | 85340 | 3.49E+05 | 88% | 0 | 99.11% | MG662131.1 |
| *Carpinus tientaiensis* chloroplast, complete genome | 86756 | 3.75E+05 | 98% | 0 | 99.01% | KY174338.1 |
| *Carpinus tientaiensis* chloroplast, complete genome | 86756 | 3.75E+05 | 99% | 0 | 99.01% | KY117036.1 |
| *Carpinus caroliniana* chloroplast, complete genome | 84958 | 3.76E+05 | 98% | 0 | 99.01% | NC_039935.1 |
| *Carpinus caroliniana* chloroplast, complete genome | 84958 | 3.78E+05 | 99% | 0 | 99.01% | MF977768.1 |
| *Carpinus fangiana* chloroplast, complete genome | 91526 | 3.61E+05 | 92% | 0 | 99.00% | MG386371.1 |
| *Carpinus cordata* chloroplast, complete genome | 92414 | 3.77E+05 | 98% | 0 | 98.99% | MF977769.1 |
| *Carpinus fargesiana* chloroplast, complete genome | 91445 | 3.67E+05 | 95% | 0 | 98.99% | NC_039937.1 |
| *Carpinus fargesiana* chloroplast, complete genome | 91445 | 3.80E+05 | 99% | 0 | 98.99% | MF977771.1 |
| *Carpinus fangiana* chloroplast, complete genome | 92374 | 3.79E+05 | 99% | 0 | 98.97% | NC_039936.1 |
| *Carpinus fangiana* chloroplast, complete genome | 92374 | 3.80E+05 | 99% | 0 | 98.97% | MF977770.1 |
| *Carpinus cordata* chloroplast, complete genome | 91491 | 3.79E+05 | 99% | 0 | 98.97% | KY312849.1 |
| *Ostrya trichocarpa* chloroplast, complete genome | 84963 | 3.71E+05 | 97% | 0 | 98.97% | KY088271.1 |
| *Carpinus oblongifolia* chloroplast, complete genome | 92202 | 3.78E+05 | 98% | 0 | 98.92% | NC_038092.1 |
| *Carpinus oblongifolia* chloroplast, complete genome | 92202 | 3.77E+05 | 98% | 0 | 98.92% | MG720817.1 |
| *Carpinus tschonoskii* chloroplast, complete genome | 92189 | 3.68E+05 | 95% | 0 | 98.92% | NC_039938.1 |
| *Carpinus tschonoskii* chloroplast, complete genome | 92189 | 3.79E+05 | 99% | 0 | 98.92% | MF977772.1 |
| *Ostrya trichocarpa* isolate Otr08 chloroplast, complete genome | 71278 | 3.55E+05 | 91% | 0 | 98.92% | MG662127.1 |
| *Carpinus polyneura* chloroplast, complete genome | 92195 | 3.68E+05 | 95% | 0 | 98.91% | NC_039998.1 |
| *Carpinus polyneura* chloroplast, complete genome | 92195 | 3.68E+05 | 95% | 0 | 98.91% | MG386373.1 |
| *Ostrya trichocarpa* isolate Otr12 chloroplast, complete genome | 71267 | 3.55E+05 | 91% | 0 | 98.91% | MG662125.1 |
| *Ostrya trichocarpa* isolate Otr11 chloroplast, complete genome | 71263 | 3.55E+05 | 91% | 0 | 98.91% | MG662126.1 |
| *Carpinus monbeigiana* chloroplast, complete genome | 92124 | 3.67E+05 | 95% | 0 | 98.89% | NC_039997.1 |
| *Carpinus monbeigiana* chloroplast, complete genome | 92124 | 3.61E+05 | 92% | 0 | 98.89% | MG386372.1 |
| *Carpinus rupestris* chloroplast, complete genome | 92078 | 3.66E+05 | 94% | 0 | 98.87% | NC_039999.1 |
| *Carpinus rupestris* chloroplast, complete genome | 92078 | 3.53E+05 | 89% | 0 | 98.87% | MG386374.1 |
| *Carpinus purpurinervis* chloroplast, complete genome | 91901 | 3.77E+05 | 98% | 0 | 98.80% | NC_038093.1 |
| *Carpinus purpurinervis* chloroplast, complete genome | 91901 | 3.77E+05 | 98% | 0 | 98.80% | MG720818.1 |
| *Carpinus viminea* chloroplast, complete genome | 91899 | 3.66E+05 | 94% | 0 | 98.80% | NC_039939.1 |
| *Carpinus viminea* chloroplast, complete genome | 91899 | 3.77E+05 | 98% | 0 | 98.80% | MF977773.1 |
| *Carpinus hebestroma* chloroplast, complete genome | 53168 | 3.78E+05 | 98% | 0 | 98.77% | NC_038131.1 |
| *Carpinus hebestroma* chloroplast, complete genome | 53168 | 3.77E+05 | 98% | 0 | 98.77% | MG720819.1 |
| *Alnus nitida* isolate 1998-654 (K) chloroplast, complete genome | 57269 | 3.58E+05 | 99% | 0 | 98.40% | MF136513.1 |
| *Alnus glutinosa* chloroplast, complete genome | 52503 | 3.11E+05 | 79% | 0 | 98.37% | NC_039930.1 |
| *Ostryopsis davidiana* chloroplast, complete genome | 81288 | 3.78E+05 | 98% | 0 | 98.05% | MH628451.1 |
| *Ostryopsis davidiana* chloroplast, complete genome | 81207 | 3.81E+05 | 99% | 0 | 98.03% | NC_039130.1 |
| *Ostryopsis davidiana* chloroplast, complete genome | 81207 | 3.82E+05 | 99% | 0 | 98.03% | MF375337.1 |
| *Ostryopsis davidiana* chloroplast, complete genome | 81166 | 3.71E+05 | 96% | 0 | 97.99% | MG386376.1 |
| *Alnus jorullensis* subsp. jorullensis isolate 2014-40 (K) chloroplast, complete genome | 62560 | 3.59E+05 | 99% | 0 | 97.99% | MF136508.1 |
| *Alnus rubra* chloroplast, complete genome | 62550 | 3.59E+05 | 99% | 0 | 97.99% | MG356709.1 |
| *Alnus rubra* isolate 1979-786 (K) chloroplast, complete genome | 62550 | 3.59E+05 | 99% | 0 | 97.99% | MF136515.1 |
| *Ostryopsis intermedia* chloroplast, complete genome | 76179 | 3.77E+05 | 98% | 0 | 97.97% | NC_040000.1 |
| *Ostryopsis intermedia* chloroplast, complete genome | 76179 | 3.77E+05 | 98% | 0 | 97.97% | MG386377.1 |
| *Ostryopsis nobilis* chloroplast, complete genome | 76112 | 3.71E+05 | 96% | 0 | 97.95% | NC_040001.1 |
| *Ostryopsis nobilis* chloroplast, complete genome | 76112 | 3.71E+05 | 96% | 0 | 97.95% | MG386378.1 |
| *Alnus japonica* isolate 1999-155 (K) chloroplast, complete genome | 62508 | 3.61E+05 | 99% | 0 | 97.95% | MF136507.1 |
| *Ostrya rehderiana* chloroplast, complete genome | 55950 | 3.76E+05 | 98% | 0 | 97.41% | MG584735.1 |
| *Fagus sylvatica* chloroplast, complete genome | 53404 | 2.31E+05 | 55% | 0 | 96.47% | NC_041437.1 |
| *Fagus sylvatica* chloroplast, complete genome | 53404 | 2.31E+05 | 55% | 0 | 96.47% | MK598696.1 |
| *Fagus crenata* chloroplast, complete genome | 53372 | 2.31E+05 | 55% | 0 | 96.44% | NC_041252.1 |
| *Fagus crenata* chloroplast, complete genome | 53372 | 2.68E+05 | 72% | 0 | 96.44% | MH171101.1 |

**Table S5** Simple sequence repeats within the cv Tombul chloroplast genome

| No | SSR type | Size | Region | Start | End |
| --- | --- | --- | --- | --- | --- |
| 1 | (GAAA)3 | 12 | LSC | 361 | 372 |
| 2 | (AAT)4 | 12 | LSC | 3061 | 3072 |
| 3 | (T)10 | 10 | LSC | 4236 | 4245 |
| 4 | (T)10 | 10 | LSC | 4712 | 4721 |
| 5 | (A)11 | 11 | LSC | 5221 | 5231 |
| 6 | (C)11 | 11 | LSC | 5507 | 5517 |
| 7 | (T)11 | 11 | LSC | 7498 | 7508 |
| 8 | (T)10 | 10 | LSC | 7734 | 7743 |
| 9 | (A)12 | 12 | LSC | 8015 | 8026 |
| 10 | (A)12 | 12 | LSC | 8491 | 8502 |
| 11 | (TCTT)3 | 12 | LSC | 9627 | 9638 |
| 12 | (TA)7 | 14 | LSC | 9827 | 9840 |
| 13 | (A)10 | 10 | LSC | 10653 | 10662 |
| 14 | (AT)6 | 12 | LSC | 11275 | 11286 |
| 15 | (GTCT)3 | 12 | LSC | 12896 | 12907 |
| 16 | (A)10 | 10 | LSC | 13455 | 13464 |
| 17 | (T)10 | 10 | LSC | 14022 | 14031 |
| 18 | (T)11 | 11 | LSC | 14162 | 14172 |
| 19 | (T)11 | 11 | LSC | 18473 | 18483 |
| 20 | (A)12 | 12 | LSC | 18633 | 18644 |
| 21 | (T)10 | 10 | LSC | 20573 | 20582 |
| 22 | (T)11 | 11 | LSC | 20701 | 20711 |
| 23 | (T)12 | 12 | LSC | 21239 | 21250 |
| 24 | (AT)5 | 10 | LSC | 22080 | 22089 |
| 25 | (T)10 | 10 | LSC | 28501 | 28510 |
| 26-27 | (C)14(A)12* | 26 | LSC | 30355 | 30380 |
| 28 | (G)13 | 13 | LSC | 30562 | 30574 |
| 29 | (T)11 | 11 | LSC | 31681 | 31691 |
| 30 | (AATA)3 | 12 | LSC | 33583 | 33594 |
| 31 | (AT)5 | 10 | LSC | 35341 | 35350 |
| 32 | (TAT)4 | 12 | LSC | 35687 | 35698 |
| 33 | (TCTA)3 | 12 | LSC | 39775 | 39786 |
| 34 | (A)12 | 12 | LSC | 40536 | 40547 |
| 35 | (A)10 | 10 | LSC | 40810 | 40819 |
| 36 | (A)10 | 10 | LSC | 46508 | 46517 |
| 37 | (T)10 | 10 | LSC | 52331 | 52340 |
| 38 | (AT)5 | 10 | LSC | 55283 | 55292 |
| 39 | (T)11 | 11 | LSC | 55574 | 55584 |
| 40 | (T)10 | 10 | LSC | 58680 | 58689 |
| 41 | (T)10 | 10 | LSC | 59165 | 59174 |
| 42 | (AACAGA)3 | 18 | LSC | 62346 | 62363 |
| 43 | (AT)5 | 10 | LSC | 63730 | 63739 |
| 44 | (T)11 | 11 | LSC | 65168 | 65178 |
| 45 | (TC)5 | 10 | LSC | 65632 | 65641 |
| 46-47 | (AT)5tattggtatattgtgattgtgtcctccagaaaaaacaaggggggggtcgagtga(TTCT)3 | 76 | LSC | 68217 | 68292 |
| 48-49 | (AT)5tattggtatattgtgattgtgtcctccagaaaaaacaaggggggggtcgagtga(TTCT)3 | 76 | LSC | 69747 | 69822 |
| 50 | (T)11 | 11 | LSC | 72990 | 73000 |
| 51 | (TA)8 | 16 | LSC | 74035 | 74050 |
| 52 | (TTTA)3 | 12 | LSC | 76645 | 76656 |
| 53 | (A)13 | 13 | LSC | 76844 | 76856 |
| 54 | (A)10 | 10 | LSC | 78274 | 78283 |
| 55 | (TA)5 | 10 | LSC | 86701 | 86710 |
| 56 | (A)10 | 10 | LSC | 87228 | 87237 |
| 57 | (TA)6 | 12 | IRB | 91109 | 91120 |
| 58 | (GCAAT)3 | 15 | IRB | 105664 | 105678 |
| 59 | (AGGT)3 | 12 | IRB | 112256 | 112267 |
| 60 | (AG)5 | 10 | IRB | 114151 | 114160 |
| 61 | (ATT)4 | 12 | SSC | 117369 | 117380 |
| 62-63 | (AAAT)3aaaaatcttttatttttaattaactgtttctgattcaccagctctta(T)11gaaaggaatcagttaataaaaaaattaaaatatataaaact(A)10 | 121 | SSC | 118886 | 119006 |
| 64 | (A)10 | 10 | SSC | 119529 | 119538 |
| 65 | (A)11 | 11 | SSC | 120076 | 120086 |
| 66-67 | (TATT)3atatttatata(AT)5 | 33 | SSC | 120291 | 120323 |
| 68 | (AT)5 | 10 | SSC | 120456 | 120465 |
| 69 | (A)12 | 12 | SSC | 120580 | 120591 |
| 70 | (C)11 | 11 | SSC | 121101 | 121111 |
| 71 | (AT)5 | 10 | SSC | 122136 | 122145 |
| 72 | (TTGA)3 | 12 | SSC | 124603 | 124614 |
| 73 | (T)10 | 10 | SSC | 126075 | 126084 |
| 74 | (TTC)4 | 12 | SSC | 128068 | 128079 |
| 75 | (AT)7 | 14 | SSC | 130508 | 130521 |
| 76 | (T)11 | 11 | SSC | 131628 | 131638 |
| 77 | (T)10 | 10 | SSC | 133593 | 133602 |
| 78 | (A)10 | 10 | SSC | 134962 | 134971 |
| 79 | (CT)5 | 10 | IRA | 137705 | 137714 |
| 80 | (CTAC)3 | 12 | IRA | 139596 | 139607 |
| 81 | (CATTG)3 | 15 | IRA | 146186 | 146200 |
| 82 | (AT)6 | 12 | IRA | 160744 | 160755 |

**Table S6** Comparison of two SSR identification tools for the cv Tombul chloroplast genome

| IMEx-web | | | | | |  | MISA | | | | | |
| --- | --- | --- | --- | --- | --- | --- | --- | --- | --- | --- | --- | --- |
| No | **SSR type** | **Size** | **Region** | **Start** | **End** |  | **No** | **SSR type** | **Size** | **Region** | **Start** | **End** |
| 30 | (TAAA)3 | 12 | LSC | 33585 | 33596 |  | 30 | (AATA)3 | 12 | LSC | 33583 | 33594 |
| - | - | - | - | - | - |  | 52 | (TTTA)3 | 12 | LSC | 76645 | 76656 |
| 72 | (TGAT)3 | 12 | SSC | 124604 | 124615 |  | 72 | (TTGA)3 | 12 | SSC | 124603 | 124614 |
| 74 | (TCT)4 | 12 | SSC | 128069 | 128080 |  | 74 | (TTC)4 | 12 | SSC | 128068 | 128079 |

**Table S7** Differences between cv Tombul and KX822768 cp genome published in GenBank

| Differences | Genes |
| --- | --- |
| Unprocessed genes in KX822768, but found in cv Tombul | *atpF*, *clpP* |
| Not found in KX822768 | *accD*, *psbM*, *trnI*-*GAT* |
| Found twice in cv Tombul | *psbF*, *psbJ*, *psbL* |

**Table S8** The features of Fagales and Malpighiales plastomes

| Species | | Size (bp) | LSC (bp) | SSC (bp) | IR (bp) | Number of protein-coding gene | Number of tRNA genes | Number of rRNA genes | GC content (%) |
| --- | --- | --- | --- | --- | --- | --- | --- | --- | --- |
| *Corylus avellana cv* Tombul | 161,667 | 90,198 | 18,733 | 26,368 | 86 | 31 | 8 | 36.40 |  |
| *Corylus chinensis* Franch | 159,915 | 88,115 | 17,822 | 26,989 | 95 | 29 | 8 | 36.49 |  |
| *Corylus fargesii* | 159,856 | 88,313 | 18,339 | 26,602 | 94 | 29 | 8 | 36.51 |  |
| *Betula nana* L. | 160,579 | 89,492 | 19,343 | 25,872 | 86 | 40 | 8 | 36.10 |  |
| *Castanea mollissima* | 160,869 | 90,497 | 18,970 | 25,701 | 86 | 37 | 8 | 36.75 |  |
| *Juglans regia* | 160,352 | 89,871 | 18,413 | 26,034 | 80 | 36 | 8 | 36.10 |  |
| *Populus cathayana* | 156,789 | 84,851 | 16,594 | 27,672 | 85 | 37 | 8 | 36.70 |  |
| *Populus schneideri* | 156,513 | 84,778 | 16,495 | 27,620 | 85 | 37 | 8 | 36.70 |  |
| *Quercus aliena* | 161,150 | 90,444 | 19,054 | 25,826 | 86 | 40 | 8 | 36.83 |  |

**Table S9** Differences between annotation tools

|  |  | Annotation Tools | | |
| --- | --- | --- | --- | --- |
|  |  | **cpGAVAS** | **Dogma** | **GeSeq** |
| Genes | ***atpF*** | No gap | One gap | One gap |
|  | ***clpP*** | No gap | Two gap | Two gap |
|  | ***ndhA*** | No gap | One gap | One gap |
|  | ***ndhB*** | No gap | One gap | One gap |
|  | ***ndhK*** | No gap | One gap | No gap |
|  | ***petA*** | No gap | One gap | No gap |
|  | ***rpl2*** | No gap | One gap | One gap |
|  | ***rpoC1*** | No gap | One gap | One gap |
|  | ***ycf3*** | No gap | Two gap | Two gap |
|  | ***ycf15*** | No gap | One gap | - |
| tRNAs | ***trnA-TGC*** | - | + | - |
|  | ***trnfM-CAT*** | + | + | - |
|  | ***trnI-CAT*** | + | + | - |
|  | ***trnI-GAT*** | - | + | + |
|  | ***trnK-TTT*** | - | + | - |
|  | ***trnL-TAA*** | - | + | - |
|  | ***trnV-TAC*** | - | + | - |

* - : not found; +: found


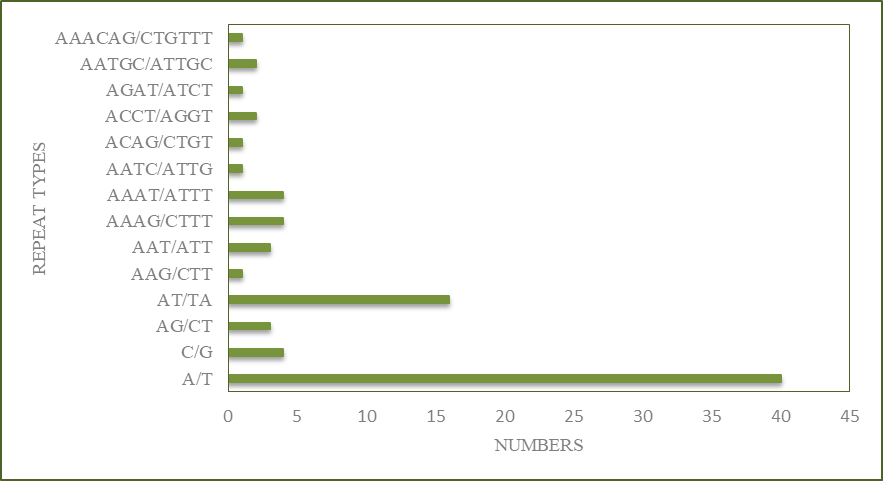


**Figure S1** Number of classified SSR repeat types (considering complementary sequences)


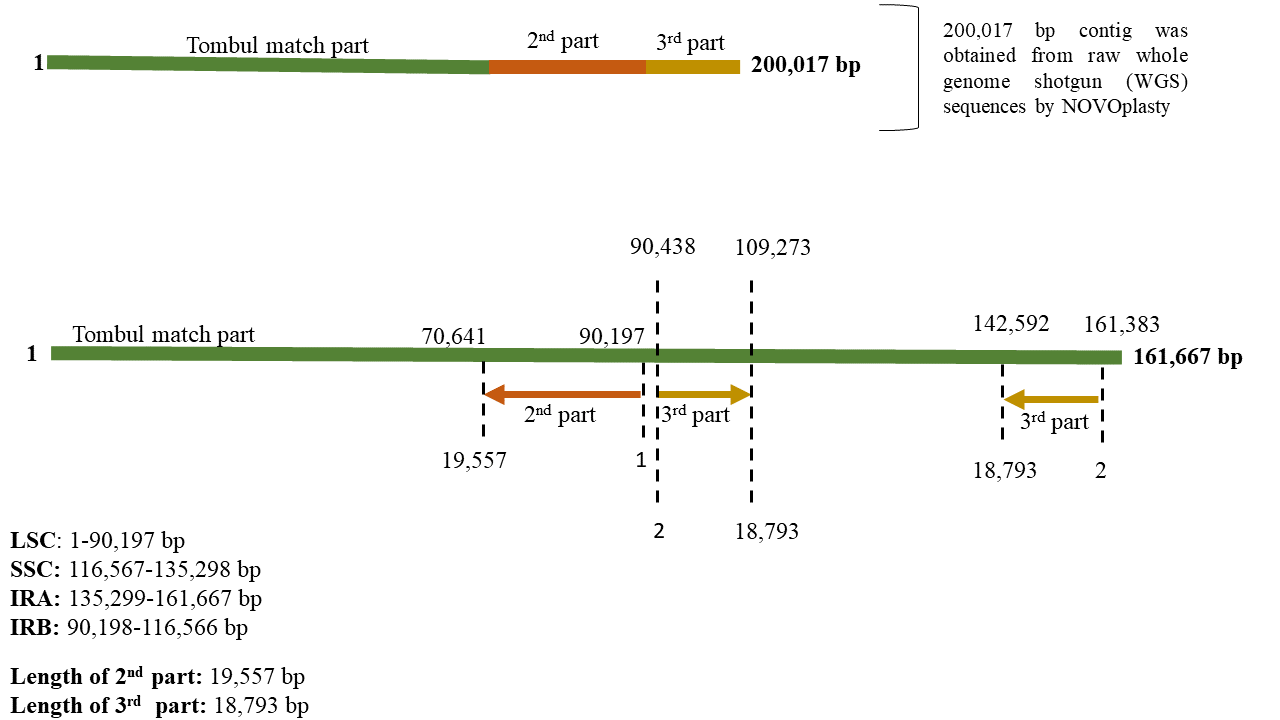


**Figure S2** Schematic that explains the structure of cv Tombul chloroplast genome
